# Multi-layered global gene regulation in mouse embryonic stem cells

**DOI:** 10.1007/s00018-014-1734-9

**Published:** 2014-09-17

**Authors:** Samuel Beck, Bum-Kyu Lee, Jonghwan Kim

**Affiliations:** Department of Molecular Biosciences, Institute for Cellular and Molecular Biology, Center for Systems and Synthetic Biology, The University of Texas at Austin, Austin, TX 78712 USA

**Keywords:** Transcriptional regulation, *Cis*-regulatory element, Enhancer, Promoter, Core pluripotency factors, MYC class DNA binding protein (DBPs), Polycomb repressive complex (PRC), Co-occupancy, Epigenetic regulation, Histone modification, Histone modifiers, DNA methylation, Chromatin remodeler, High-order chromosomal structure, Long-range chromosomal interaction, Chromosomal territory, Modular transcriptional regulation, Protein–protein interaction (PPI), Protein–DNA interaction (PDI)

## Abstract

Embryonic stem (ES) cells derived from the inner cell mass of developing embryos have tremendous potential in regenerative medicine due to their unique properties: ES cells can be maintained for a prolonged time without changes in their cellular characteristics in vitro (self-renewal), while sustaining the capacity to give rise to all cell types of adult organisms (pluripotency). In addition to the development of protocols to manipulate ES cells for therapeutic applications, understanding how such unique properties are maintained has been one of the key questions in stem cell research. During the past decade, advances in high-throughput technologies have enabled us to systematically monitor multiple layers of gene regulatory mechanisms in ES cells. In this review, we briefly summarize recent findings on global gene regulatory modes in ES cells, mainly focusing on the regulatory factors responsible for transcriptional and epigenetic regulations as well as their modular regulatory patterns throughout the genome.

## Introduction

Embryonic stem (ES) cells are in vitro cultural counterparts of the inner cell mass (ICM, mouse) [1] or epiblast (human) [2] of developing embryos. These cells self-renew for a prolonged time in vitro while keeping their potential to generate all three germ layers (pluripotency) [3]. Due to these unique characteristics which are not observed in their in vivo counterparts, ES cells have attracted tremendous attention as useful tools for studying early mammalian development, making genetically-modified mouse models to unravel gene functions [4], and nurturing future therapeutic applications in regenerative medicine.

During the early era of mouse ES cell studies, establishments of ES cells were limitedly successful in only few mouse strains [3, 5]. Therefore, ES cells were often regarded as cultural artifacts until ES cells from other species/strains were later reported [3]. However, the limitations in deriving ES cells due to the initial strain dependency forced various studies to be performed on almost identical genetic backgrounds and culture conditions, allowing diverse data sets from independent experiments to be easily integrated for systematic analyses. Except for a few human cell lines extensively tested by the ENCODE project [6, 7], mouse ES cells are the most widely studied mammalian model system.

Over the last decade, understanding the underlying regulatory mechanisms enabling the unique characteristics of ES cells has been one of the major goals in the field of stem cells and developmental biology [3, 8]. Besides the uniqueness of ES cells, accumulated knowledge of transcriptional regulations in ES cells significantly advanced our view of mammalian gene regulation. In particular, recently developed high-throughput methodologies, such as massive-parallel sequencing [9], enabled researchers to perform unbiased mappings of chromosomal targets of many transcription factors (TFs), DNA-binding proteins (DBPs), and epigenetic regulators, as well as epigenetic modifications including DNA and histone modifications. These efforts tremendously expanded our understanding of the regulatory mechanisms of global gene expression programs in ES cells where various regulatory entities are sophisticatedly interconnected to sustain the unique identities of ES cells.

In this review, we provide an overview of recent advances in understanding global gene regulatory mechanisms in mouse ES cells. We summarize key regulatory factors and their roles in transcriptional and epigenetic regulations. Another focus is on global gene regulations mediated by long-range interactions among multiple chromatin domains in ES cells. We also discuss ES cell-specific modular regulations where functionally separable sub-networks are tightly intertwined to maintain self-renewal and pluripotency of mouse ES cells.

## Transcriptional regulation in es cells

As one of the key regulatory mechanisms determining cellular characteristics, transcriptional regulation is mediated by multiple components including *cis*-regulatory elements (promoters, enhancers, and insulators) and *trans*-acting factors (sequence-specific TFs, general TFs, co-activators [10], co-repressors, mediators [11], chromatin modifiers, and chromatin remodelers [12, 13]). The importance of transcriptional regulation was recently highlighted by master TFs-mediated cell fate changes, such as somatic cell reprogramming [14] and trans-differentiation [15–19].

### Core pluripotency TFs in ES cells

In ES cells, Oct4, Sox2 and Nanog have been traditionally considered the core pluripotency TFs. The core TFs are exclusively expressed in early embryos and play critical roles in maintaining ES cell identity by governing the ES cell-specific gene expression program [20]. They were initially identified in loss-of-function studies, and an individual disruption of *Oct4* [21], *Sox2* [22] or *Nanog* [23] abolished early embryogenesis due to the failures of forming intact ICM, where mouse ES cells are derived, indicating their central roles in establishing and maintaining the pluripotency of ES cells. Proper levels of the core TFs in ES cells are important for both maintaining pluripotency and suppressing differentiation. Niwa et al. [24, 25] showed that approximately twofold induction of Oct4 in mouse ES cells prompts mesodermal and endodermal differentiation, while 50 % reduction of Oct4 results in differentiation toward a trophectoderm (TE) lineage by inducing Cdx2 and Eomes. Moreover, a recent study by Radzisheuskaya et al. [26] shows the reduced level of Oct4 in ES cells results in the failure of normal differentiation into embryonic lineages, while restoration of Oct4 rescues the differentiation capability. Consistently, Oct4^+/−^ ES cells can maintain stabilized pluripotency state accompanying with increased Oct4 occupancy across the genome, but shows compromised differentiation due to the delay in initial exit from the ESC state [27]. These results indicate that the level of Oct4 in wild-type ES cells is necessary for proper differentiation into all embryonic lineages. Similarly, the knockdown of Sox2 in ES cells leads to primarily TE differentiation [28, 29], whereas ectopic expression of Sox2 induces multiple lineages [30]. The similar phenotypic consequences upon perturbations of Oct4 or Sox2 imply a functional linkage between these two master TFs. Consistently, Oct4 and Sox2 form a heterodimer and synergistically activate their pluripotency-related target genes, including Nanog [31] and Fgf4 [32]. Unlike Oct4 and Sox2, an elevated level of Nanog was sufficient to maintain mouse ES cells without leukemia inhibitory factor (LIF), and Nanog-deficient ICM was prone to differentiate into parietal endoderm-like cells and fail to form an intact epiblast [23, 33]. However, further study performed by Chambers et al. [34] showed that, although they are prone to differentiate, Nanog null ES cells can self-renew infinitely in vitro, colonize embryonic germ layers, and contribute to the somatic lineages of fetal and adult chimaeras. Further studies suggested that pluripotency-related TFs are not only critical in ES cell maintenance but also function as lineage-specifying factors [35–37].

Initial efforts to understand global target gene regulation by the core TFs were made using human [20] and mouse [38, 39] ES cells by combining chromatin immunoprecipitation (ChIP) and microarray (ChIP-chip) or paired-end tag sequencing (ChIP-PET), revealing that the core TFs share a substantial number of common target genes including core TFs themselves [20]. These core pluripotency TFs-mediated auto-regulatory and feed-forward regulatory mechanisms suggested that the core TFs form a tight regulatory circuit to maintain ES cells. More recently, ChIP analyses followed by massive-parallel sequencing (ChIP-seq) uncovered that the core TFs co-occupy mainly distal enhancer regions rather than promoters of target genes [40] to form pluripotency-specific enhanceosomes in mouse ES cells [41–43].

### Extended core regulatory network in ES cells

To gain more insights into the detailed action mechanisms of the core pluripotency TFs, pull-downs of protein complexes followed by mass spectrometry analyses were performed, and various interaction partner proteins of the core TFs were identified [44–49]. Orkin and colleagues [44] conducted a pioneer work to define a Nanog-centered protein–protein interaction (PPI) network and found multiple previously unknown Nanog-associated proteins, including Nacc1 (Nac1), Zfp281, Dax1, Sall4, and Rif1. From subsequent pull-downs of newly-identified Nanog-associated proteins they constructed an extended Nanog-centered PPI network [44]. Interestingly, depletion of Nanog-associated factors in ES cells often showed the loss of pluripotency, suggesting that many Nanog-associated TFs may play critical roles in the maintenance of ES cells. A more recent study has newly identified eight additional Nanog-interacting partners, including Tet (10–11 translocation) family proteins, which synergistically enhance somatic cell reprogramming with Nanog [50]. Oct4-centered PPI network was also defined by multiple independent research groups; van den Berg et al. [46] showed that Oct4 is physically associated with 166 proteins, including TFs (Sall4, Tcfcp2l1, Dax1, and Esrrb) and chromatin modifiers (SWI/SNF and NuRD complexes) that are implicated in the self-renewal of ES cells. Depletion of Oct4 decreased the chromosomal target occupancy of Oct4-associated factors, indicating that Oct4 recruits its associated factors and cooperatively regulates their common targets. Another pull-down study of Oct4 by Pardo et al. [47] also revealed that Oct4 interacts with a wide range of TFs and epigenetic regulators. The mutations of Oct4-interacting partners often lead to the early lethality of developing embryos, indicating that Oct4-associated factors also exert critical roles in early embryogenesis. Although there was an apparent discrepancy between Oct4-centered interactomes defined by these two independent groups, most recent mapping of Oct4 interaction partners by Ding et al. [48] identified larger size (~198 proteins) of high-confident Oct4-interactome with significant overlap with both of two previous studies [46, 47]. All these elements indicate that surprisingly many factors are involved in the regulation of ES cells in addition to the three core pluripotency TFs.

In addition to the PPI studies, an extended core TF-centered protein–DNA interaction (PDI) network was constructed using a ChIP-based method. Global target mapping of four somatic cell reprogramming factors (Oct4, Sox2, Klf4, and cMyc) [14] and some Nanog-associated TFs (Nanog, Dax1, Rex1, Zfp281, and Nacc1) [44] revealed that target genes bound by few factors tested are in general inactive, whereas common targets of many TFs are mostly active in ES cells [39]. The results suggest that there might be a differential gene regulatory mode relying on target co-occupancy of regulatory TFs. This observation also raises the question of how pluripotency factors discriminately activate or repress their target genes. One possible explanation is that multiple ES cell core TFs and their associated factors form enhanceosomes by co-occupying enhancers to promote target gene activation [40], while Oct4 and Nanog also form a distinctive repressive complex such as NODE (Nanog and Oct4-associated deacetylase), containing HDAC1/2 (histone deacetylases 1/2) and Mta1/2 [45]. Induced expression of development-related genes upon knockdown of the NODE complex subunit supported the idea that the NODE is responsible for the repression of lineage-specific marker genes in ES cells [45].

### Modular action of various TFs

**Fig. 1:**
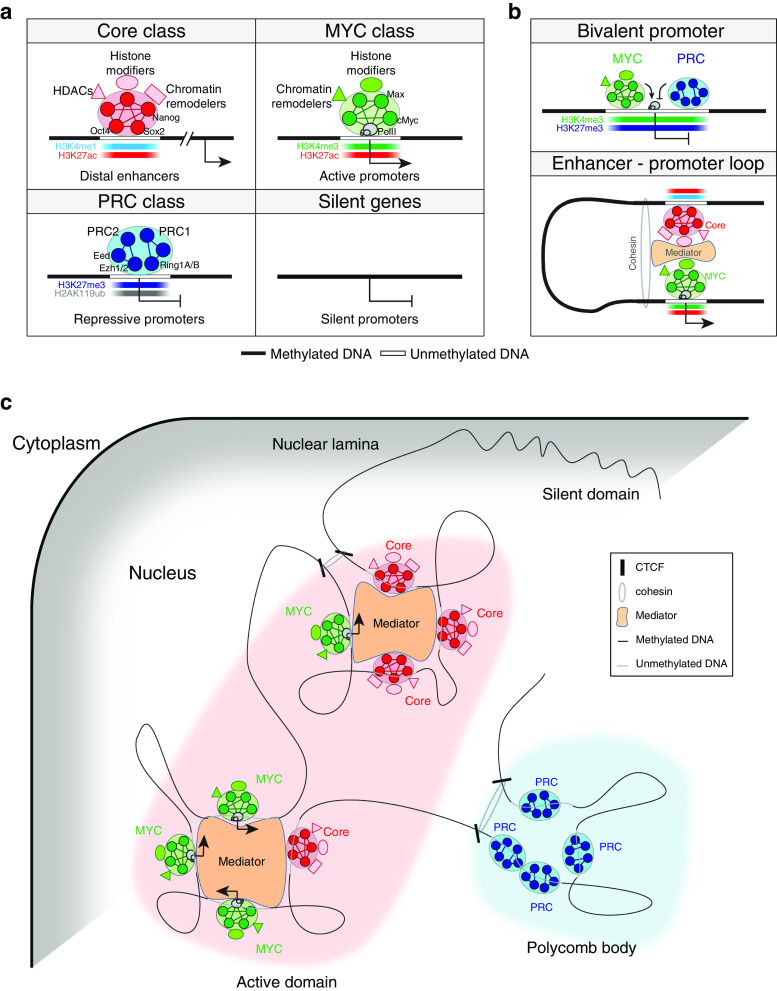
Schematic representation of global gene regulatory modes in ES cells. **a** Transcriptional and epigenetic regulatory classes defined in ES cells. Recent studies of mapping targets of TFs and histone-modifying enzymes as well as histone modification signatures revealed that tested factors belong to mainly three classes based on their target co-occupancy (Core, MYC and PRC classes) [51]. As depicted, each class is associated with distinct TFs/DBPs, *cis*-regulatory elements and histone modification marks. Notably, core pluripotency factors including Oct4 and Nanog belong to the Core class and occupy distal enhancer elements with enhancer specific histone modification marks (H3K4me1 and H3K27ac). Both MYC and PRC classes occupy proximal promoters, but show opposite target gene activities with unique histone modification marks (MYC class: H3K4me3 and H3K27ac; PRC class: H3K27me3 and H2AK119ub). Regulation of silent genes under the control of methylated promoters has not been well-understood in ES cells. Detailed factors, histone marks and other information involved in each regulatory class are summarized in Table 1. **b** Interactions between regulatory classes in ES cells. Proximal promoters of development or lineage specification-related genes that are mainly repressed in ES cells harbor bivalent histone marks (H3K4me3 and H3K27me3), and are associated with both MYC and PRC classes (*upper panel*). Distal enhancer elements occupied by the Core class factors interact with the MYC class-bound proximal promoters via a long-range chromosomal looping. Interactions between two classes are facilitated by mediator and cohesion complex (*lower panel*). **c** Spatial compartmentalization of chromosomal domains with regulatory classes. Active chromatin domains formed in the center of nucleus show tight interconnection between the Core and MYC classes via long-range chromosomal interactions. Genes repressed by the PRC class are co-localized and form nuclear sub-compartments called polycomb bodies. The repressive polycomb bodies are distinct from the nuclear lamina-associated silent domains anchoring at the nuclear periphery. Ctcf and cohesion demarcate chromatin domains

While unbiased mapping of targets of various TFs in ES cells disclosed their cooperative actions on common target gene regulations [20, 39, 40, 51], differential target occupancy patterns were observed between the targets of the core TFs and targets of cMyc [40, 51]. The results indicated that TFs in ES cells can be divided into multiple distinct classes based on their target occupancy patterns. Recent analysis of the chromosomal targets of many active TFs in ES cells defined three functionally separable TF classes (Core, MYC and PRC; Table 1; Fig. 1a) [51]. The core pluripotency TFs, Oct4, Sox2 and Nanog, as well as other TFs, such as Smad1, Stat3, Nacc1, Dax1, and Zfp281, form the Core class TFs, and mainly co-occupy distal regulatory elements of mouse ES cells. On the other hand, cMyc, its binding partner proteins (Max, Tip60 and Dmap1), and other TFs sharing their targets with cMyc (Zfx, Cnot3, E2F1, and E2F4) form the MYC class and co-occupy mainly promoter elements of highly active genes in ES cells. The PRC class is composed of factors in polycomb repressive complexes 1 and 2 (PRC1 and PRC2, respectively), and occupies promoters of inactive genes, including lineage-specific regulators in ES cells. In the pluripotent state of ES cells, targets of the Core and MYC classes are highly active while targets of the PRC class are inactive [51]. Upon differentiation, targets of the Core and MYC classes are suppressed whereas targets of the PRC class are induced. Interestingly, target gene activities of the Core and MYC classes were opposite in partially reprogrammed cells [51], suggesting that each class of TFs may form a functionally separable regulatory subnetwork.

**Table 1:** Distinct transcriptional regulatory classes in mouse ES cells

Entity	Class/module			
	Core class	MYC class	PRC class	Silent genes
TFs/DBPs (regulators)	Core pluripotency factors [20] (Oct4, Sox2, Nanog) Core interacting partners [44, 46–48, 50] (Nacc1, Zfp281, Dax1, Sall4, Rif1, Sall4, Tcfcp2l1, Esrrb) Other enhancer binding TFs [39, 40] (Smad1, Stat3, Nacc1, Dax1, Zfp281)	Myc [40, 51] (cMyc, nMyc) Myc binding partners [51] (Max, Tip60, Dmap1) Other promoter binding TFs [51] (Zfx, Cnot3, E2F1, E2F4)	Canonical PRC1 [57, 105] (Cbx7, Ring1b) Non-canonical PRC1 [106–108] (Rybp, Ring1b) PRC2 [57, 105] (Ezh2, Suz12, Jarid2, Eed2)	Unknown (Absent?)
Target *cis*-regulatory element	Enhancer [40, 54]	Promoter [51]		–
Distance from transcription start site	Distal	Proximal		–
Activities of target genes in ES cells [51]	Active		Inactive	Silent
Histone modification	H3K4me1, H3K27ac (active enhancer), H3K27me3 (poised enhancer) [121, 122]	H3K4me3, H3K27ac [51]	H3K27me3, H2AK119ub [57, 105]	–
Functions of target genes [101]	Pluripotency	Cellular metabolism	Development	Physiological response
Subnuclear location	Center [151]		Center (polycomb bodies) [170]	Periphery [152]
Chromosomal interaction	With MYC enriched promoters and other Core enriched enhancers [166, 167]	With Core enriched enhancer and other MYC enriched promoters [166, 167]	With other PRC enriched promoters [170]	With Nuclear lamina [152]
Chromosomal interaction proteins	Coactivators [163], Mediators [53, 54], Klf4 [168]		Eed [170]	–
	Ctcf, cohesion [160–162]			
Associated chromatin remodelers	Brg1 [128], Chd3/4 [124], Chd7 [141]	Tip60–p400 complex [51, 130], INO80 [132], Bptf [135], Chd1 [137]	–	–
Associated histone modifiers	MLL3/4 complex [190], p300 [40], HDACs, Lsd1 [124, 186]	Tip60–p400 complex [51], SET1/MLL1-2 complex [114]	PRC1, PRC2 [57, 105]	–
Activities of target genes upon differentiation [51]	Decrease		Increase	Inert

The core class TFs largely co-occupy distal enhancer elements of pluripotency-related genes with a previously known enhancer binding protein p300, a histone acetyltransferase [52], and form ES cell-specific enhanceosomes [40] to promote communication between enhancers and promoters via protein-mediated long-range interactions (Fig. 1b). Accordingly, Young and colleagues [53] reported that mediator proteins such as Med1 and Med12 share common targets with multiple core TFs. With extremely strong mediator occupancy signals, they further defined ‘super-enhancers’ spanning large domains of chromatin [54]. Their works additionally suggested that the context-dependent conformation of super-enhancers bound by tissue-specific master TFs and mediators primarily determine the cell-type specific gene expression program. Interestingly, most recent study of Oct4 and Sox2 using single molecule imaging analyses in ES cells has revealed that Sox2 binds first to target enhancers followed by the recruitment of Oct4 [55], suggesting that the core TFs assemble enhanceosomes in a hierarchical order. Notably, the Core class includes downstream effectors in signaling pathways, such as Smad1 and Stat3 [40], further supporting the notion that enhancers function as integration hubs of external signaling [56].

Unlike the Core class, ChIP-seq analyses revealed that the MYC and PRC classes preferentially occupy the proximal promoters of their target genes [51, 57]. Given that cMyc interacts with multiple proteins, including a NuA4 (nucleosome acetyltransferase of H4) complex in ES cells, and cooperatively regulates a common set of target genes, how the factors composing the NuA4 complex influence the pluripotent state of ES cells will be of great interest. Recently, two independent research groups reported that an elevated level of cMyc amplifies the expression of its already existing target genes instead of de novo activation of additional target genes [58, 59], suggesting that cMyc functions as a universal nonlinear amplifier for all active genes rather than a binary on–off switching factor. In agreement with this observation, Rahl et al. [60] showed that cMyc plays a key role in releasing paused RNA polymerase II (PolII) via its interaction with positive transcription elongation factor b (P-TEFb) rather than recruiting PolII at its target genes. Although all these studies support that cMyc globally functions as a transcriptional activator, it also can, with Miz1 and/or other co-repressor proteins such as Hdac1, suppress the expression of differentiation-related genes, including Hox cluster genes in human ES cells [61–64]. Future studies will be required to determine the mechanistic roles of cMyc as a repressor and its global contribution to promote the pluripotent state of ES cells.

Mapping unbiased targets of TFs is critical for understanding the roles of specific TFs. However, even with various individual and group efforts, less than 10 % of DBPs within the genome have been subjected to ChIP-seq study in mammalian cells [7]. Since the majority of factors so far tested in ES cells belong to only a few classes, one obvious question is whether there are TFs forming unique subnetworks other than the three classes so far reported in ES cells [51]. The lack of ChIP-grade antibodies for a broad range of TFs has been an obstacle in mapping TF occupancies even with multiple systematic efforts to catalog ChIP-grade antibodies [65–67]. Alternative attempts, such as tagging TFs, have been tried to circumvent current limitations and to increase throughput [39, 68–70]. Considering the complexity of the global transcriptional regulation in which hundreds of DBPs are tightly interconnected with numerous *cis*-regulatory elements, further systematic studies of all active TFs in ES cells, possibly with more advanced technologies, will be necessary to gain more comprehensive insights into the pluripotency-specific global transcriptional regulatory mechanisms in ES cells.

## Epigenetic regulation in ES cells

In addition to transcriptional regulation, structural alterations of chromatin by epigenetic regulations including covalent modifications of DNA and histone tails, as well as alterations of nucleosome positions, render a favorable or unfavorable environment for TFs to interact with *cis*-regulatory elements of the genome, and eventually modulate a specific gene expression program. Together with TFs, elaborate and dynamic interplay among various epigenetic regulators play pivotal roles in maintaining the pluripotent state of ES cells as well as in determining proper cell fates during development.

### The roles of DNA methylation in pluripotency of ES cells

The first layer of epigenetic regulation is DNA methylation, which primarily occurs at the cytosine of a CpG dinucleotide, producing 5-methylcytosine (5mC). In mammals, approximately, 70–80 % of total CpG sequences in their genomes are methylated [71], while some *cis*-regulatory regions, such as active enhancers or promoters are hypomethylated in a context-dependent manner [72]. Another type of DNA elements called ‘CpG islands’ (CGI), where multiple CpG dinucleotides are clustered and stretched near promoters [71], show mostly constant hypomethylation regardless of the tissue types or developmental stages [73, 74]. Among three well-conserved DNA methyltransferases (DNMTs; Dnmt1, Dnmt3a and Dnmt3b), Dnmt3a and Dnmt3b are responsible for de novo methylation during early embryogenesis [75]. On the other hand, Dnmt1 maintains genomic methylation during cell divisions. Deletion of both Dnmt3a and Dnmt3b blocks de novo methylation in ES cells, resulting in developmental abnormalities [76]. Similarly, homozygous deletion of Dnmt1 leads to delayed development followed by early embryonic lethality [77]. Notably, Dnmt1-null ES cells are capable of self-renewing in vitro [77], but die during differentiation due to increased apoptosis [78]. Overexpression of Dnmt1 also results in embryonic lethality due to the loss of imprinting caused by global hypermethylation of genome [79].

Although methylated DNA is generally considered a signature for gene silencing, some studies suggested that de novo methylation does not induce silencing of active promoters [80]. Therefore, the precise mechanisms involved in DNA methylation-mediated gene silencing still remain to be determined. One plausible model is that DNA methylation passively hampers the binding of TFs as most TFs preferentially bind to the regions lacking DNA methylation [81]. For example, the promoters of *Oct4* and *Nanog* are hypomethylated in ES cells; however, CpG dinucleotides in those promoters become hypermethylated along with complete silencing of their expressions upon differentiation [82, 83]. Another model involves methyl-binding domain containing proteins (Mbds), which can recognize methylated DNAs and further interact with other repressor complex proteins [84]. Nucleosome-remodeling deacetylase (NuRD) complex, a well-known repressor complex comprising HDAC1, HDAC2, Mi-2α/β (also known as Chd3/Chd4), RbA and Mta [84–87], binds to its target through Mbd proteins that can recognize methylated DNA [84].

Recently, important roles of Tet proteins (Tet1, Tet2 and Tet3), which can convert 5mC to various demethylated forms of DNA, such as 5-hydroxymethylcytosine (5hmC), 5-formylcytocine (5fC) and 5-carboxylcytosine (5caC), have been illuminated in ES cells [88, 89]. In particular, Tet1 sustains the level of Nanog in ES cells by maintaining consistent demethylation of *Nanog* promoters in ES cells [90]. Tet1/Tet2 proteins also physically interact with Nanog and facilitate somatic cell reprogramming by establishing naïve pluripotency through their catalytic activity [50]. Moreover, Tet1 replaces Oct4 during somatic cell reprogramming via reactivation of Oct4 by demethylation of the *Oct4* promoter [91]. Genome-scale mapping studies also revealed that Tet1 preferentially occupies CGI to prevent undesirable activity from DNMTs [92], contributing to the establishment of poised chromatin status, which is evidenced by the strong enrichment of 5hmC, particularly in the proximal regions of transcription start sites harboring both active (H3K4me3) and repressive (H3K27me3) histone marks (see below) [93]. Conversely, other studies showed that Tet1 single- or Tet1/Tet2 double-knockout (DKO) ES cells maintain pluripotency [94, 95]. Although a portion of Tet DKO embryos died before birth, apparently normal Tet mutant mice with slightly reduced levels of 5hmC, as well as some aberrant methylation, raises speculation that Tet3 may compensate for the loss of Tet1 and Tet2. The individual Tet proteins need to be further characterized to clarify the roles of DNA methylation status in ES cells as well as during early developments.

### Promoter-specific epigenetic regulations

Histone tails of eukaryotes are often covalently-modified with acetylation, methylation, phosphorylation, and ubiquitination, encompassing diverse information on local chromatin statuses, favorable for either activation or repression of related genes (histone code, [96]). Studies revealed that the chromatin architecture of ES cells is globally open, marked with abundant active histone signatures including H3K4me3 and acetylation-enriched histones, and is transcriptionally hyperactive [97, 98]. In conjunction with these observations, the majority of promoters in ES cells harbor active histone marks, such as H3K4me3, H3K9ac and H3K14ac, accompanied by preloaded PolII [99]. These histone signatures are associated with transcription initiation, but only subsets of genes are transcribed into full-length mRNA with elongation histone marks (H3K36me3).

Interestingly, prior works showed that lineage-specific genes in ES cells harbor very distinct histone signatures called ‘bivalent marks’ (Fig. 1b) [100]. The repressed lineage-specific regulators have bivalent histones containing both active (H3K4me3) and repressive (H3K27me3) marks that are rapidly activated upon differentiation. Genes with bivalent marks or repressive mark (H3K27me3 only) in their promoters are functionally distinct from non-marked genes (without H3K4me3/H3K27me3) that are silent in ES cells (Table 1; Fig. 1a) [101, 102]. Intriguingly, promoters of the core TFs, such as Oct4, Sox2 and Nanog, are switched from active (H3K4me3) to bivalent marks when ES cells undergo differentiation, revealing that histone modifications are not associated with a specific functional category of genes, but reflect the context-dependent activity of genes [101].

The two most well-known histone marks (H3K27me3 and H3K4me3) are generally observed near the proximal promoters, and generated by PRC and Trithorax-group (TrxG) proteins, respectively [103, 104]. Polycomb complexes, PRC1 and PRC2, primarily suppress developmental regulators in ES cells [57, 105] by forming H2AK119ub and H3K27me3, respectively. Recently, studies of CxxC domain containing proteins involved in the PRC-mediated repression revealed the sequential recruitment of PRCs to their repressive target genes. Briefly, CxxC containing Kdm2b occupies CpG promoters and recruits non-canonical PRC1 (Rybp-PRC1), priming a repressive histone mark (H2AK119ub), which in turn recruits PRC2, generating a H3K27me3 mark. Subsequently, canonical PRC1 (Cbx7-PRC1) is recruited to further expand H2AK119ub mark [106–108]. Interestingly, deletion of subunits in PRC2 did not affect the self-renewal of ES cells [57, 105, 109, 110]. However, PRC2-depleted ES cells showed apparent defects in differentiation, suggesting the important roles of PRC in the proper differentiation of ES cells [110, 111].

The active histone mark H3K4me3 is catalyzed by TrxG group proteins such as Set1A/B or Mll1/2 (mixed lineage leukemia 1/2), forming a SET1/MLL complex [104, 112]. Wdr5, a core member of the SET1/MLL complex, physically interacts with Oct4, and its depletion impairs self-renewal of ES cells and somatic cell reprogramming [113]. In contrast, loss of Dpy-30, another subunit of the SET1/MLL complex, abrogates pluripotency while maintaining self-renewal of ES cells [114]. Notably, the occupancy of TrxG group proteins is largely guided by MYC class TFs such as Max, as Max interacts with Wdr5 in ES cells [51] and in HeLa cells [115]. Mof, another MYC family protein, also plays an imperative role in sustaining ES cells by recruiting Wdr5 to active promoters via a physical interaction [116]. These observations provide strong evidence of the collaborative modular regulation of the MYC class TFs, TrxG proteins, and corresponding active histone signatures (Table 1).

### Enhancer-specific epigenetic regulations

Enhancers act over a long distance and enhance the activity of target gene promoters to govern the identity of specific cell types by connecting tissue-specific master TFs, mediators, and RNA PolII machinery [117]. The formation of enhancer-TFs complexes (enhanceosomes) is context-specific, as suggested in the interaction between core pluripotency TFs and ES cell-specific distal regulatory elements [40]. Recent genome-wide studies revealed that the global enhancer connectivity within the genome is extensively reorganized to change tissue-specific gene expression programs during differentiation [54, 118–120]. Although a conventional definition of enhancers was linked to their interaction with transcriptional co-activators [117], additional characteristics of enhancers, such as p300 occupancy and a prevalent H3K4me1 mark, have been reported and have become widely used for the annotation of tissue-specific enhancer elements [56, 119, 121, 122]. Furthermore, more recent studies classified enhancers in ES cells into two groups (active and poised enhancers) depending on the combination of multiple histone marks [119, 121, 122]. Active enhancers are generally open with low nucleosome density and marked with both H3K4me1 and H3K27ac signatures with bindings of p300 and Brg1, a subunit of the SWI/SNF chromatin remodeling complex [119, 121, 122]. On the other hand, poised enhancers are functionally inert in self-renewing ES cells with H3K27me3 signatures, but rapidly acquire active enhancer signatures upon differentiation.

For successful differentiation of ES cells, active enhancers controlling ES cell-specific genes must be inactivated. Histone H3K4/K9 demethylase Lsd1 (also known as Kdm1a), the first mammalian histone demethylase identified [123], has been reported to localize at the active enhancers in ES cells and modulate the inactivation of ES cell-specific enhancers [124]. While Lsd1-depleted ES cells could self-renew normally in vitro, the cells showed defects in differentiation mainly due to the failure of histone demethylation at ES cell-specific enhancers. Other studies suggested that Lsd1 interacts with the NuRD complex, suggesting that other factors are also required for proper differentiation [124]. Consistently, ES cells lacking Mbd3, a subunit of NuRD complex, failed to silence the activity of pluripotency factors upon differentiation, and these cells self-renew even in the absence of LIF [125]. Interestingly, global target mapping of HDACs and NuRD complexes revealed that they are more enriched in the enhancers of active genes rather than repressed genes [124, 126], which is somewhat inconsistent with our general understanding of their repressive enzymatic functions. It is conceivable that HDACs may play a major role in attenuating the expression of active genes to balance their levels within an appropriate range, or they may prime active genes for future repression during differentiation.

### Influence of chromatin remodeling on pluripotency of ES cells

ATP-dependent chromatin remodelers provide open or closed chromatin structures by rearranging nucleosome compositions or repositioning nucleosomes [12, 13]. These local chromosomal changes, along with other histone and DNA modifying enzymes in either a conjunctive or sequential manner, affect the accessibility of transcriptional machineries onto *cis*-regulatory elements, and eventually determine local gene activity. ATP-dependent chromatin remodeling complexes can be divided into several groups, such as SWI/SNF, ISWI, and CHD, with different types of ATPase core subunits [13].

ES cells have a distinctive SWI/SNF complex so-called esBAF comprised of Baf155, Baf60A, Baf250a, and the ATP-dependent helicase Brg1 [127, 128]. Depletion of Baf250a resulted in defects, particularly in mesodermal lineage specifications upon differentiation of ES cells [129]. Brg1 co-localizes with the core TFs on pluripotency-specific enhancers [128], implying its involvement in maintaining low nucleosomal density at enhancers. Another SWI/SNF complex, Tip60-p400, physically interacts and shares global targets with cMyc at active promoters harboring H3K4me3 marks in mouse ES cells [51, 130]. p400 has been known to replace H2A with histone variants such as H2A.Z in the nucleosome of active promoters [131]. ES cells with depletion of Tip60 or p400 showed reduced proliferation, up-regulation of lineage-specific genes, defects in embryoid bodies (EB), and teratoma formations, indicating that Tip60-p400 is required for maintaining the identity of ES cells [130]. A recent study additionally revealed that INO80, another SWI/SNF chromatin-remodeling complex, is also critical for maintaining ES cell identity [132].

Mammalian ISWI ATP-dependent chromatin-remodeling complexes containing Snf2h or Snf2l ATPase facilitate the sliding of nucleosomes by disrupting the interactions between histone proteins and DNA [133]. The importance of Snf2h was shown by the embryonic lethality of Snf2h-null mice before implantation due to the defects in cell growth of the blastocyst stage embryo [134]. A study of another ISWI complex NURF (nucleosome remodeling factor) containing Snf2l and Bptf (bromodomain and PHD finger TF) revealed that Bptf is associated with H3K4me3 marks [135]. Bptf-null ES cells showed deregulation of genes implicated in development of all three germ layers and genes particularly regulated by Smad, suggesting that Bptf links a Smad signaling pathway to the transcription of lineage-specific genes [136].

Chromodomain helicase DNA-binding (CHD) family chromatin remodelers also have been known to play critical roles in sustaining pluripotency. Chd1 recognizes H3K4me2/3 [137] and occupies the promoters of active genes where the MYC class TFs bind [51, 138]. Chd1-deficient ES cells self-renew in vitro but are preferentially differentiated into neural lineages upon EB formation while showing defects in primitive endoderm differentiation [139]. Interestingly, these cells also showed accumulation of heterochromatin, suggesting that Chd1 plays direct roles in rendering open chromatin to prevent heterochromatin formation in ES cells [139]. A disruption of another CHD family, Chd7 in mouse embryos resulted in prenatal death due to the multiple tissue defects [140]. In ES cells, Chd7 co-localizes with the core factors on active enhancers [141] and plays a role as a molecular rheostat to maintain the level of ES cell-specific factors. As discussed earlier, Chd3/4, also known as Mi-2α/β in NuRD complex, were also reported to occupy active enhancers in ES cells [124].

## Higher-order chromatin architecture

So far, we discussed the factors responsible for the transcriptional and epigenetic regulations in ES cells and their functions occurring through local changes in chromatin structures of the genome. More recently, studies have revealed that chromatin structures are further organized into specific higher-order architectures depending on cell types and developmental stages [142]. A growing body of evidence supports that higher-order chromatin structures also play an imperative role as a new regulatory layer controlling stem cell characteristics. In this section, we summarize recent understandings of higher-order chromatin architectures mediated by long-range interactions.

### Unique and dynamic chromatin structures of ES cells

The chromatin structure of an ES cell is largely open with less heterochromatin composition, loosely compacted compared to the chromatin of differentiated cells, and transcriptionally hyperactive due to active chromatin-remodeling enzymes and general TFs [97, 143, 144]. The chromatin plasticity of ES cells is believed to secure rapid genomic adaptation upon differentiation to promote lineage-specific gene expression programs [145, 146]. The recently developed Hi-C assay [147], a powerful tool in identifying global higher-order chromatin interactions, suggested that chromatins are largely organized with distinct megabase-sized topological domains [148]. This is consistent with previous microscopic observations of subcellular gene locations showing that gene-enriched chromosomal domains are located in the center of the nucleus, while gene-depleted regions or centromeres are found in the nuclear periphery (Fig. 1c) [149, 150]. Chromosomes of human ES cells showed similar organization wherein pluripotency genes, such as Nanog and Oct4, are located closer to the center of the nucleus [151]. Upon differentiation, repressive histone modifications are elevated globally while pluripotency genes are relocated from the center of the nucleus to the nuclear lamina to become silenced [152]. These results suggest that the genomic structures of ES cells are not only established to provide a favorable environment for maintaining ES cell identity, but also experience reorganization to benefit specific lineage commitments during differentiation.

### Techniques for chromosomal conformation capturing

Early studies of chromatin conformations relied on microscopic observation via DNA fluorescence in situ hybridization (FISH) for a limited number of genomic loci. Chambeyron et al. [153] showed that the extrusion of a *Hoxb* locus from its chromosomal territories coincides with its activation. This observation brought up the idea that chromosomal localization may affect gene activity. Thanks to the development of chromosome conformation capture (3C) technology, local chromatin structures and small-scale physical interactions between distant genomic regions have been studied in depth [154, 155]. To identify multiple interacting regions of a given genomic locus, chromosome capture has been integrated with circularization (circular chromosome conformation capture: 4C) or other genome-wide analysis tools, such as tiling array (4C-array or chromosome conformation capture-on-chip [156]) and high-throughput parallel sequencing (4C-seq [157]). Additional approaches were invented to capture chromosomal interactions within a cell as a whole (carbon-copy chromosome conformation capture: 5C [158], Hi-C [147]). More recently, a new method was developed to study unbiased chromosomal interactions mediated by specific proteins (chromatin interaction analysis by paired-end tag sequencing: ChIA-PET, for the review of technological details, see [155]).

### Long-range interaction mediated higher-order chromatin structures in ES cells

It has been shown that long-range interactions and higher-order chromatin structures are mediated by specific proteins [159] such as nuclear Ctcf/cohesin [160–162], p300/Cbp [163], and mediators [164, 165], and these proteins link one chromosomal region to another via long-range looping. An integrative analysis using HI-C and Ctcf ChIP-seq in human cells revealed that Ctcf is a major architect building chromosomal structures by mediating interactions both within a chromosome and between different chromosomes (Fig. 1c) [161]. ChIA-PET assays of Ctcf in mouse ES cells identified 1,480 *cis*- and 336 *trans*-acting chromatin interactions [160], and another study on Ctcf-mediated loops unveiled that Ctcf partitions distinct chromatin compartments with different transcriptional and epigenetic statuses [162]. Ctcf loops also determine the boundaries of lamin-associated silent chromosomal regions and function as a barrier between silent and active regions [162].

Additionally, ChIA-PET assays of PolII disclosed interactions between multiple active promoters (P–P interactions) and pervasive interactions between promoters and enhancers (P–E interactions) [166, 167]. Notably, promoters of the core TFs (*Oct4*, *Sox2*, *Nanog,* and *Klf4*) are interconnected within close physical proximity in ES cells. However, in neural stem cells, *Sox2* gene is connected with *Olig1* and *Olig2* genes that play important roles in decision of neural cell fate, implying that gene relocation and replacement of interaction partners coincide with differentiation. On the other hand, regulatory elements of *Oct4* gene lacking PolII-mediated chromatin interactions in somatic cells obtain intrachromosomal interactions during somatic cell reprogramming [166], highlighting critical roles of long-range looping-mediated chromatin structure changes in pluripotency-specific gene expression programs. Notably, unlike a prior assumption of proximity-governed interactions between enhancers and promoters, this work revealed that approximately 75 % of enhancers communicate with distal promoters rather than their nearest promoters.

Mediators are major culprits in linking promoters and enhancers as a complex with cohesin, bridging two different chromosomal regions by encircling them with cohesin rings [53]. Depletion of these proteins in ES cells results in overall collapse of ES cell-specific chromatin interactions and loss of a pluripotent state. Moreover, as discussed earlier, extremely high levels of mediator occupancy are associated with super-enhancers that regulate a global tissue-specific gene expression program, supporting the importance of long-range interactions mediated by mediators [54]. Other co-activators, such as p300 and Cbp, also mediate long-range looping at *Nanog* locus in ES cells by physical interaction with Nanog proteins, in turn activating other pluripotency genes [163].

The fact that pluripotency factors such as Oct4 and Nanog particularly bind to the enhancers of ES cell-specific genes suggested that pluripotency factors may also play important roles in organizing chromatin configurations. A recent 4C-seq study has revealed that Klf4 proteins recruit cohesin onto the *Oct4* enhancer before the activation of endogenous Oct4 during somatic cell reprogramming [168]. Similarly, depletion of Klf4 leads to differentiation of ES cells due to the disruption of Klf4-mediated long-range interactions between *Oct4* enhancers and promoters, indicating that Klf4 also involves high-order chromatin architecture to induce pluripotency [168]. Another 4C-seq study of *Nanog* locus also emphasized the interactions between *Nanog* locus and other pluripotency-specific genes [169]. Importantly, Eed-mediated long-range looping at the PRC-mediated repressive regions also has been reported [170]. Particularly, genes repressed by PRC are co-localized together within distinct nuclear sub-compartments called polycomb bodies [171] that are different from previously known silent nuclear peripheries (Table 1; Fig. 1c) [170].

## Summary and future perspectives

### Interconnection of multiple regulatory mechanisms

Research in the past decade aided by modern systems biology tools have demonstrated that the global gene expression program in mouse ES cells is controlled by multiple layers of regulatory steps, such as transcriptional and epigenetic regulations. Emerging patterns of tightly interconnected regulatory pathways are more evident with recent demonstrations of long-range interactions among various *cis*- and *trans*-regulatory elements.

Epigenetic signatures generated by the recruitment and action of histone modifiers [52, 172, 173] and chromatin remodeling complexes [12, 87] are intertwined with transcriptional regulation mediated by sequence-specific and general TFs. De novo bindings of TFs to their target sites are affected by pre-formed local nucleosome density [174, 175], DNA methylation status [81], epigenetic modifications [172], and other preoccupied transcription factors [176]. Sequence information in *cis*-regulatory elements becomes functional only when they are occupied by regulatory factors with accompanying chromatin and epigenetic statuses. Now it is clear that these associations between epigenetic modifications and TF occupancies are collectively formed within separate chromosomal territories [148] with long-range interactions [166]. Table 1 summarizes interrelations between each transcriptional regulatory class so far known in ES cells and the entities of global gene regulation, including TFs, epigenetic regulators, epigenetic modifications, chromatin status, subcellular localizations, and so on. All these regulatory layers and regulators should be collectively considered as a whole, not as separate entities, for a more comprehensive understanding of pluripotency-specific gene expression program.

### Implications of modular transcription and epigenetic regulations

Notably, analysis of accumulated TF or DBP occupancy data suggested that a group of TFs or regulators tend to share similar chromosomal targets as a functionally separable regulatory subunit as shown in mouse ES cells: the Core, MYC and PRC classes [51]. The co-occupancy mediated modular action of TFs seems to help transcriptional controls in diverse ways. First, the cooperation of multiple TFs within each class may secure the successful execution of gene regulation compared to the sole action of a single TF. Conversely, each TF within a subnetwork may have unique functions to make up a complete functionality, as Tcf3, a negative regulator of transcription, shares common targets with the core factors in ES cells and balances the levels of the core factors [177]. Second, more importantly, TFs or DBPs within a subnetwork are almost always associated with distinct histone-modifying enzymes and histone modification marks, as summarized in Table 1 and Fig. 1, suggesting that the gene expression programs in ES cells is achieved by the modular actions of both TFs and epigenetic regulators. This view is particularly imperative, as suggested in the works by Zaret and colleagues [176, 178–180]; pioneering factors recognize and occupy target sequences in an epigenetic status-independent manner at the beginning, and then recruit other TFs or epigenetic regulators, allowing changes in chromatin status, further interacting with other *cis*-regulatory elements to form higher-level chromatin interactions, in turn changing cellular characteristics [167, 179, 181]. Third, the modular actions of TFs and epigenetic regulators seem to ensure rapid and precise cellular responses in environmental changes, as shown in Lsd1 and Mi-2/NuRD cases [124]. As discussed above, these putative repressors are responsible for the removal of pluripotency-specific enhancers upon differentiation, implying their roles in rapid response to environmental changes. Last, the modular action of multiple factors also involves signal transduction pathways [40]. As most effectors of signaling pathways are TFs, their actions are dependent on the pre-formed modular regulatory network, such as enhanceosomes [40]. This also explains previous observations of context-specific responses of signaling pathways [182–184].

### Implication of chromosomal interactomes

Recently attained knowledge on chromatin conformation in ES cells suggested important biological significances. Considering limited cellular resources, spatial segregation of active, repressive and silent regions may enable more efficient way of gene regulation [170]. In addition, as described above, chromosomal conformations not only constrain global gene expression, but also involve gene activity changes in response to environmental changes or differentiation cues [166, 185].

Moreover, results from the enhancer-promoter interactome studies disclosed that multiple enhancers interact not only with a single promoter, but frequently with multiple promoters to form clusters of multiple co-expressed genes [166, 167]. More collective and high-ordered approaches to map global chromosomal interactomes will be required for the acquisition of more comprehensive views of global gene regulatory mechanisms.

### Future directions

To our surprise, some of the newly-attained genome-wide data seemed somewhat inconsistent with our conventional understanding of the functions. For example, the genomic targets of some previously known repressor proteins such as Lsd1 [124, 186] and Hdac1 [126] are near the regulatory elements of active genes rather than the repressed genes in ES cells. Moreover, general approaches of mapping TF targets often do not show any strong correlation with the activity of the target genes upon perturbation of tested factors [187–189]. Therefore, to get better insights into the roles of TFs or epigenetic regulators, in addition to their enzymatic functions and context-dependent genome-wide occupancies, the interacting partners, target *cis*-elements and three-dimensional chromatin interactions should be considered together. Aligned with these comprehensive studies linking diverse aspects of gene regulations, conventional genetics studies aiming to understand the functions of each regulatory factor in early development or in vivo should also be continued.

## Abbreviations

ES cell: Embryonic stem cell
ICM: Inner cell mass
DBP: DNA-binding proteins
TF: Transcription factor
TE: Trophectoderm
LIF: Leukemia inhibitory factor
ChIP: Chromatin immunoprecipitation
PET: Paired-end tag
ChIP-seq: Chromatin immunoprecipitation followed by massive-parallel sequencing
PPI: Protein–protein interaction
PDI: Protein–DNA interaction
NODE: Nanog and Oct4-associated deacetylase
NuA4: Nucleosome acetyltransferase of H4
CGI: CpG island
DNMT: DNA methyltransferase
Mbd: Methyl-binding domain containing protein
NuRD: Nucleosome remodeling deacetylase
DKO: Double knockout
H3K36me3: Histone H3 lysine 36 trimethylation
H3K4me1: Histone H3 lysine 4 monomethylation
H3K4me3: Histone H3 lysine 4 trimethylation
H3K27me3: Histone H3 lysine 27 trimethylation
H3K27ac: Histone H3 lysine 27 acetylation
H2AK119ub: Histone H2A lysine 119 ubiquitination
EB: Embryoid body
CHD: Chromodomain helicase DNA-binding
FISH: Fluorescence in situ hybridization
3C: Chromosome conformation capture
4C: Circular chromosome conformation capture
5C: Carbon-copy chromosome conformation capture
ChIA-PET: Chromatin interaction analysis by paired-end tag sequencing
PolII: RNA polymerase II
